# The intersection of genomics and big data with public health: Opportunities for precision public health

**DOI:** 10.1371/journal.pmed.1003373

**Published:** 2020-10-29

**Authors:** Muin J. Khoury, Gregory L. Armstrong, Rebecca E. Bunnell, Juliana Cyril, Michael F. Iademarco

**Affiliations:** 1 Office of Genomics and Precision Public Health, Office of Science, Centers for Disease Control and Prevention, Atlanta, Georgia, United States of America; 2 Office of Advanced Molecular Detection, National Center for Emerging and Zoonotic Infectious Diseases, Centers for Disease Control and Prevention, Atlanta, Georgia, United States of America; 3 Office of Science, Centers for Disease Control and Prevention, Atlanta, Georgia, United States of America; 4 Office of Technology and Innovation, Office of Science, Centers for Disease Control and Prevention, Atlanta, Georgia, United States of America; 5 Center for Surveillance, Epidemiology and Laboratory Services, Centers for Disease Control and Prevention, Atlanta, Georgia, United States of America

## Abstract

Muin Khoury and co-authors discuss anticipated contributions of genomics and other forms of large-scale data in public health.

**Summary points**

- The field of precision public health (PPH) has emerged as a response to the increasing availability of genomics, biobanks, and other sources of big data in healthcare and public health.
- The field has evolved starting with genomics to include multiple practical applications such as pathogen genomics that address population health.
- PPH can expand understanding of health disparities, advance strategic public health science, and demonstrate the need for innovation and workforce development.
- In the coronavirus disease 2019 (COVID-19) era, rapidly evolving scientific innovation can have a long-lasting impact on PPH beyond the pandemic.
- Further developments in PPH will require global, national, and local leadership and stakeholder engagement.

## Introduction

In the past few years, precision public health (PPH) has emerged as a multidisciplinary field [1,2] that relies on big data and data science [3] to drive public health assessment, policy, and implementation activities. The use of the word “precision” in the context of population-level activities as opposed to individualized precision medicine interventions has generated a variety of reactions [4–7]. The word is traditionally applied to individuals and may seem at odds with public health efforts focused on improving population health. In addition, as the field of precision medicine has so far identified mostly with genomic medicine, there was concern about its silence on social and environmental determinants that are important drivers of population health. An online dialogue and a paper elaborated on the tension between precision medicine and population health and the need for both personalized and population-level interventions to improve health [8]. Increasingly, big data offer opportunities for wider implementation of PPH approaches together with methodological strategies to address potential limitations.

Recently, a special series of articles explored the topic of PPH in *Frontiers in Public Health* [9]. In their introductory editorial, Weeramanthri and colleagues [10] discussed how the term was introduced in Western Australia as a way to use genomics, spatial technology in health, and data linkages to complement the developments in “precision medicine,” a term used in a 2011 US National Research Council Report [11]. In 2015, the US government launched its precision medicine initiative [12], focusing on cancer and the development of a large cohort of a million or more participants for a longitudinal study of health and disease (AllofUs Research Program) [13]. In 2016, the concept of PPH was introduced in the peer-reviewed literature as a call to modernize surveillance and information systems, as well as targeted interventions from a population health perspective [1]. In 2019, we elaborated on the notion of PPH as much larger in scope than individual genomic variation [14], as well as the need to use novel data sources and analytics to modernize public health tracking and implementation by time, place, and persons [14]. While we recognize that PPH is a global issue, national public health systems and their approaches do vary significantly. Therefore, in this review we focus mainly on the US health and public health system to demonstrate important points and examples.

## From genomics and big data to precision medicine and PPH

In the past decade, we have seen an explosion in health- and non-health-related data. With electronic health records, genomic and other biomarkers, and emerging use of wearable biomonitoring devices, the sheer amount of information available for population analysis is large and increasing exponentially [2]. Perhaps as opposed to “small data,” big data are “too complex and varied to be contained in one spreadsheet or sometimes even in one computer” [15]. Big data can be integrated from many sources related to people, places, and time, such as geographic information systems, electronic health records, digital biomarkers, online apps, and social media.

Banks [15] recently reported some staggering numbers about big data. Examples include an estimated worldwide health data of 2,314 exabytes (10^18^ bytes) in 2020 and 25 petabytes (10^15^ bytes) of genomic data by 2030. A single typical hospitalization generates about 150,000 pieces of data, and the market for wearable devices that can capture health related is projected to be at $16.3 billion in 2022 [15].

Big data enable the potential for more “precision” in medicine and public health. In theory, more data at the individual level can help redefine the meaning of healthy and the progression from health to disease, helping to uncover preventable disease risk factors and allowing more precision diagnostic and prognostic information. At the population level, big data can help integrate multiple social and environmental risk factors such as air pollution, neighborhood walkability, and access to healthy food.

Table 1 summarizes our take on the evolving definitions in medicine and public health in relation to how genomics and “precision” have influenced these fields. It is important to acknowledge at the outset that addressing health problems in a population requires the application of individual medical approaches [16] and population-level approaches [17,18]. Improving the health of populations is not only about delivering the most optimal healthcare but is about working across all determinants of health, ranging from genetic and biological factors to social and environmental determinants of health. The completion of the human genome project led to a new era of genomic medicine [19] driven by genetic information in individuals. In parallel, the field of public health genomics arose as an approach to use genomic information to improve the health of populations. However, the recent evolution of genomic medicine to include other types of information led to the rise of “precision medicine,” which seeks to personalize medical interventions at the individual level. PPH is more than the application of genomics in populations. Beyond genomics, all kinds of data can be used in measuring determinants of health and impact of interventions. If precision medicine is about delivering the right intervention to the right individual at the right time, PPH can be simply viewed as delivering the right intervention to the right population at the right time. Put in another way, PPH is “about using the best available data to target more effectively and efficiently interventions of all kinds to those most in need” [7].

**Table 1:** Genomics, precision medicine, and PPH: Definitions and comparisons.

	Medicine	Public Health
	**Medicine** is the art and science of practice for diagnosing, treating, and preventing disease. [16]	**Public health** is “the science of protecting and improving the health of people and their communities.” [17] What we as a society do collectively to ensure the conditions in which people can be healthy [18]
**Genomics**	**Genomic medicine** is an “emerging medical discipline that involves using genomic information about an individual as part of their clinical care.” [19]	**Public health genomics** is a multidisciplinary field concerned with the responsible and effective translation of genome discoveries into improved population health.” [20]
**Precision**	**Precision medicine** is “a novel approach to treatment and prevention that takes into account differences in lifestyle, environment, and biology.” [12] The right intervention to the right patient at the right time.	**PPH** is a novel approach that uses big data science and technology to improve population health and reduce health disparities. The right intervention to the right population at the right time.

**Abbreviation:** PPH, precision public health

## Current applications in PPH

There are several current or near-term potential applications of genomics and big data in PPH, including a focus on modernizing public health surveillance, development of targeted interventions to implement effective interventions to improve health and reduce health disparities, the use of machine learning in public health, and special applications of pathogen genomics in the public health response to infectious diseases. Recent studies, commentaries, tools, and applications in PPH can be searched in the curated and continually updated Centers for Disease Control and Prevention (CDC) Public Health Genomics and Precision Health Knowledge Base [20] for specific health conditions or public health issues.

### Public health surveillance in the era of big data

An emerging priority for public health is the use of information technology (IT) and data science in enhancing public health surveillance. Surveillance has been defined as the systematic, ongoing collection, management, analysis, and interpretation of data to stimulate and guide action [1]. Broadly, surveillance includes traditional case counting as well as surveys, registries, real-time monitoring systems (such as used by the National Syndromic Surveillance Program), and specialized studies. As recently reviewed by Dolley [21], big data’s most cited use in public health is to improve disease surveillance and “signal detection.” The richness of emerging data by place, persons, and time has the potential to accelerate public health surveillance and early disease detection and community health issues. Below are some examples.

#### Place

Using small-area analysis, we might be able to uncover pockets of health disparities that are often masked in analyses performed on areas such as counties or states. For example, a local-burden-of-disease analysis of child mortality under the age of 5 years across 46 African countries [22] showed that when mortality was analyzed at a high spatial resolution, new maps showed major disparities in child mortality even though overall progress was reported at the country level. Another example is the use of neighborhood deprivation metrics that can assess health outcomes and disparities within regions (for example, affluent towns may have pockets of health deprivation) [23]. Another example is a recent study [24] of census tracts across the US that classified social determinants of health measures using indices of advantage, isolation, opportunity, and migrant cohesion and accessibility. The analysis was conducted by 7 neighborhood typologies, which included an extreme poverty group. Social determinants of health indices were associated with premature mortality rates. This and other studies using geospatial approaches can quantify social determinants of health more precisely than using single indicators and thus could capture better the underlying complexity and heterogeneity of these factors. Thus, more “precision” in geographic, community, and health system analysis can pinpoint how best to target interventions to reduce morbidity in difficult-to-reach subpopulations and thus help reduce disparities.

More geographic precision can allow for public health resources to be used more efficiently. An example discussed by Dowell and colleagues [2] is that the *Aedes aegypti* mosquitoes that transmit dengue, Zika, and chikungunya viruses are unlikely to spread these infections if they carry a benign bacterium, *Wolbachia*. *Wolbachia*-carrying mosquitoes can be used to displace *A*. *aegypti* and reduce viral transmission, but the wide global prevalence of these viruses could make this approach costly. Using disease prevalence information and geospatial modeling has identified with more precision pinpointed areas that can serve as best candidates for introducing *Wolbachia* as a public health intervention, which can address 90% of mosquito-borne disease burden.

#### Persons

Similarly, big data are allowing a more in-depth assessment of health outcomes and disparities according to characteristics of patients and providers, beyond the use of traditional indicators such as age, sex, and race/ethnicity. Biomarkers, including genomics, are increasingly used to stratify disease outcomes and susceptibility into subgroups that reflect underlying heterogeneity and potential response to interventions. Public health surveillance systems, such as cancer registries, are benefiting from more precise diagnostic classification of cancers using biomarkers of etiology and treatment response [25].

Another example of personal heterogeneity is cholesterol education as a public health effort. A one-size-fits-all campaign could miss the diagnosis of individuals with familial hypercholesterolemia (FH), a genetic disorder affecting 1 in 250 people that remains largely undiagnosed and requires more intense identification; high-intensity low-density lipoprotein (LDL)-lowering drugs; and cascade screening in families [26]. FH represents a prototype for PPH as it points to the need to ascertain a high-risk population subgroup of a million or more individuals in the US that need aggressive treatment and cascade screening in families [26].

#### Time

Most population surveys rely on cross-sectional measurements of health indicators and risk factors. Big data may also improve precision through analysis of repeated measurements of the same variables over time. The use of personal devices such as wearable sensors and smartphones [27] can provide measurement of variability over time for various health indicators such as nutrition, physical activity, and blood pressure. Smartphones (and devices) are used increasingly to deliver evidence-based interventions (e.g., diet and nutrition programs, psychotherapy, and exercise). The data, when ethically collected through digital devices, may complement existing public health efforts in measuring population health outcomes and disparities. A recent study evaluated the use of wearable technologies to monitor the rate of influenza-like illness (ILI) at the population level [28]. As acute infections can cause an individual to have an elevated resting heart rate (RHR) and change in daily activities, the study evaluated whether population trends of seasonal respiratory infections, such as influenza, could be identified through wearable sensors that collect RHR and sleep data. The results indicated that data from wearable devices significantly improved ILI predictions over baseline models.

### Public health implementation science and strategies in the era of big data

Big data may also provide insights for the conduct of the next generation of implementation research that seeks to accelerate the translation of evidence-based discoveries into improved population health [14]. Below are some current examples of how big data could be used, by place, persons, and time, to accelerate implementation strategies of proven health interventions and address health disparities in population subgroups.

#### Place

Implementation studies can evaluate costs, effectiveness, and efficiency of interventions in real-world settings in the contexts of communities and health systems, with the goal of delivering interventions optimally across populations. The use of machine learning and decision support tools are increasingly adapted to specific health systems. Engelgau and colleagues recently discussed how tools of big data analytics can help identify barriers and facilitators for optimal implementation of interventions within the contexts of health and community policies, health systems, and community resources [29].

One specific example is the successful use of big data and analytics in pharmacy-based medication management to identify patients at highest risk for medication noncompliance or adverse effects [30]. Big data also offer important opportunities for assessing environmental and neighborhood-level factors that can increase vulnerability of populations, including density of tobacco and liquor retailers, walkability, environmental exposures, and affordable housing availability [31].

#### Persons

To reach subpopulations with unique health conditions, targeted intervention strategies will be needed. For example, although evidence-based recommendations exist for breast cancer and colorectal cancer screening for the “average” population, they do not apply to the 2 million or more individuals in the US with hereditary cancers that confer increased risk of colorectal cancer (Lynch syndrome) and hereditary breast and ovarian cancer due to BRCA mutations (HBOC) [32]. For both HBOC and Lynch syndrome, more “precision” evidence-based guidelines exist to reduce the burden of cancer in affected persons and their relatives. In the US, current public health implementation activities focus on translating and implementing these recommendations through the combination of surveillance, epidemiologic and health services research, communications, and partnerships. More recently, the National Cancer Moonshot Initiative has funded several implementation research projects to evaluate approaches in different populations and health systems for identifying and providing care for individuals with inherited cancer syndromes and their relatives [33].

#### Time

Smartphone and online apps can use big data to allow real-world analysis of health indicators over time for evidence-based interventions (e.g., medication adherence). For example, in a recent paper, a randomized clinical trial of adults with poorly controlled hypertension demonstrated that patients using a smartphone app with repeated measures showed an improvement in reported adherence to medication use [34]. Under current guidelines, all individuals in the population are subject to identical blood pressure thresholds to determine hypertension treatment. But a one-size-fits-all intervention will require the treatment of a large number of persons to prevent cardiovascular health events over time. Using longitudinal data on blood pressure facilitated by new technologies, the next generation of implementation research will allow estimates of intervention effectiveness tailored to each person, or subgroups of individuals with similar genetic, behavioral, and environmental profiles.

Big data drawn from social media are also increasingly used not only to enhance public health surveillance and detection of outbreaks but also to facilitate communication and behavior change in disease prevention and control. A recent review assessed the progress and promise of social media interventions that could enhance PPH [35]. They cautioned about how little we know about the health impact of such interventions and the risks of unintended consequences.

## Data science: Machine learning, predictive analytics, and health disparities

An important scientific foundation for the use of big data in PPH is the emerging potential for using novel forecasting and predictive analytic methods. Under the rubric of data science, novel approaches to complex analysis are now increasingly used to integrate data from many areas to arrive at more precision in measuring health and disease outcomes, which in turn can be used to improve forecasting and prediction, aiding in decision-making for resource allocation and implementation strategies. Machine learning denotes a general approach to the processing of big data, to learn patterns in the data, and validate patterns for decision makers (e.g., these approaches can be deployed to doctors, patients, or policy makers) [36].

One example that demonstrates the potential of machine learning to improve the accuracy of disease diagnosis comes from medical image analysis, such as automating screening for diabetic retinopathy [37]. Patients with diabetes are at increased risk of eye disease. Manual analysis of image data is currently a rate-limiting step that can slow down screening for eye disease in diabetes. A new branch of machine learning—deep learning—has emerged and promises to enhance health care decision-making with automated image analysis.

Cancer management and prevention provide examples [38] of important current applications for the use of big data analytics. One example is the combination of data from multiple sources (e.g., DNA germ line and tumor sequencing, gene expression, epigenetics, proteomics) along with clinical patient information and environmental exposures [39] to determine individualized cancer therapeutic strategies. A specific application of machine learning analytics is in breast cancer screening. A recent study [40] developed a machine learning approach to identify breast cancer using mammograms in large UK and US data sets. The study compared the system’s cancer predictions and clinical radiologists’ original decisions based on biopsy-confirmed breast cancer. In the US data set, the approach led to 5.7% fewer false positives and 9.4% fewer false negatives than radiologists. In the UK data set, the results were more mixed. While promising, this machine learning approach will require further replication and prospective evaluation in multiple populations.

Overall, so far, data forecasting and predictive analytics have not provided better quantitative risk prediction models than classic statistical methods such as logistic regression analysis [41]. There are several methodologic issues that need to be addressed before big data analytics can be used in PPH. These include unrepresentative or selected populations, data inaccuracy and missing information, measurement issues, and substantial concerns about confounding and inference about causality. Other issues include deficiencies in model calibration and the lack of or insufficient data sharing [42].

As discussed in a recent commentary [43], predictive analytics need to have a clear purpose. The current literature contains studies on machine learning approaches that have undergone retrospective testing but not prospective evaluation and validation. As a result, the current applications of machine learning to healthcare systems remain limited. These limits also apply to public health activities that are concerned with measuring disease- and health-related information in population subgroups outside the healthcare delivery system.

One important limitation of machine learning is its potential impact on health disparities. An important tenet of PPH is to reduce health disparities by using big data to quantify health-related outcomes in subpopulations and use this knowledge to reduce health disparities [2]. However, there are current methodologic deficiencies such as systematic bias that could result in prediction models inadvertently contributing to a widening of health disparities, especially in racial and ethnic minority populations. For example, recent studies have consistently shown that genetic risk prediction models based on genome-wide association studies are less accurate in non-European populations. This is because most genome-wide association studies have been conducted in populations of European descent [44].

Another recent example [45] is a study of a machine learning risk assessment tool that is widely used in US healthcare organizations that has erroneously assigned a large proportion of Black patients to the same level of health risk as White patients. The authors estimated that racial bias can reduce the number of Black patients identified for extra care by more than half. Bias is present because the machine learning algorithm uses health costs as a proxy for healthcare needs. As less healthcare resources are spent on Black patients who have the same level of need, the algorithm falsely concludes that Black patients are healthier than equally sick White patients. This is a real example of biased predictive analytics that can widen existing health disparities in the population. In PPH, we need to have a strong emphasis on preserving privacy and confidentiality, as well as continuously evaluating the ethical, legal, and social implications of big data and predictive analytics.

The limitations of predictive analytics are even more pronounced in the context of global health data due to incompleteness and limitations of data for measuring exposures, health outcomes, and implementation challenges around the world [46]. In this context, resource constraints can influence the development and applications of these methods. In addition, issues of fairness, accountability, transparency, and privacy preservation provide important challenges to ensure that the promise of big data and predictive analytics to improve health and reduce health disparities does not lead to unintended effects.

The goal of big data analytics is to improve decision-making both in the clinical setting and in the population context. Providing outcome probabilities may or may not change physician, patient, or health system behavior. We need to evaluate the balance between health benefits and potential harms of specific implementation interventions that use big data analytics [47].

## Pathogen genomics and PPH

Since the advent of next-generation genomic sequencing, pathogen genomics has rapidly transformed infectious disease public health, allowing for more precise detection and investigation of outbreaks, providing insights into disease emergence and transmission, enabling more accurate and efficient phenotyping of microorganisms, and thus providing more information for a PPH response, as compared with existing technologies [48,49].

Molecular subtyping of pathogens has played an important role in infectious disease public health for decades. Subtyping of pathogens into finer groups often makes outbreaks easier to detect, for example, and provides data to support or refute suspected transmission of a pathogen [48,50,51]. Almost all of these legacy subtyping technologies depend on changes in very small parts of the pathogen’s genome. Pulsed-field gel electrophoresis (PFGE), for example, produces a gel pattern that reflects polymorphisms (i.e., small changes in the genetic code) in perhaps two-dozen sites within a 5-million nucleotide bacterial genome [52]. Multi-locus sequence typing provides sequence data on only perhaps 7 of 3,000 genes in that genome. These technologies are generally very pathogen specific, may vary from laboratory to laboratory, and are usually uninformative about function.

Next-generation sequencing (also called high-throughput sequencing) increases by multiple orders of magnitude the amount of DNA that can be sequenced, allowing for the first time routine whole-genome sequencing of most pathogens. This not only enables extremely fine subtyping of those pathogens but also provides information about function—the antimicrobial resistance of a bacterial isolate, for example, or the presence or absence of a virulence factor that may affect clinical or public health decisions [52]. These data are inherently standardized. Moreover, the technology is applicable across the entire spectrum of microbes of public health importance, leading to a convergence of subtyping methods not just for bacteria but also for viral and eukaryotic pathogens [48].

In many high-income countries, next-generation sequencing is first being adopted in the bacterial foodborne disease domain, improving surveillance for such pathogens as *Salmonella*, *Campylobacter*, and *Listeria* [52]. The extremely fine subtyping provided by whole-genome sequencing allows prompt detection of outbreaks, usually signaled by the sudden emergence of a group of pathogens with identical or near-identical sequences. The technology improves investigation of those clusters by more accurately segregating outbreak from non-outbreak cases, allows for more precise confirmation of suspected food vehicles, and is a valuable tool in trace-back investigations. The switch from legacy technologies (mostly PFGE) to whole-genome sequencing in both the UK [53] and the US [54] has increased both the number of clusters of disease detected by roughly 2-fold and the number outbreaks solved. All of this is leading to a clearer picture of how pathogens are entering the food system and how they can be prevented.

The use of whole-genome sequencing in tuberculosis control is similar: extremely fine subtyping allows for detection of clusters that might otherwise be invisible [55]. Another example is Legionnaire’s disease—cluster of disease can be more confidently linked to sources such as cooling towers. In addition, the technology is providing insights into the ecology of the pathogen in water systems—insights that could eventually lead to better prevention [5]. For seasonal influenza, the paradigm is different: next-generation sequencing is accelerating characterization of the virus while providing a high-resolution picture of the dynamics of viral emergence, information that is now being used to inform vaccine strain selection [56]. Other areas of public health impacted by genomics include HIV, healthcare infection control, antimicrobial resistance monitoring, viral hepatitis outbreak surveillance and investigation, vaccine-preventable disease control, and many others [49].

## Looking ahead: Prospects for PPH

Our expectations for big data to lead to more precision in public health depend on the continued ability to modernize the use of data in healthcare and public health, the conduct of rigorous studies to evaluate the validity and utility of new data-driven approaches, and the need for innovation in applications of data science and workforce development to help integrate data science in public health.

### Data modernization in healthcare and public health

To achieve PPH, we need data modernization in healthcare. Two perspectives need to be reconciled: insurance-based priorities focused on payments and measurement of population health outcomes. The science of measuring outcomes is nascent and requires substantial research, new partnerships, and—importantly—new data.

New data will require new relationships, bringing together existing data in new ways, and collecting novel data that we don’t have either in healthcare or public health, as well as non-health data. To make progress, public health data systems need modernization and more collaboration among healthcare systems [57,58]. In turn, inter-digitation of public health data systems with healthcare systems requires robust attention.

It is important to consider the data lifecycle in healthcare, public health, and their intersection. For example, traditional public health surveillance systems at the state level collect cases of reportable conditions (such as tuberculosis and most cancers) and electronically notify the CDC, which collates and analyzes the data. After years of insufficient investment, these systems are being made interoperable. However, the data moving into the state systems need to be digitalized: many still are using phone, fax, paper reports, and incompatible digital formats. The emergence of the electronic health record presents a tremendous but challenging opportunity to bring healthcare data into public health directly. The Digital Bridge partnership and its first use case, electronic case reporting [59], presents a vision for end-to-end flow of data. The enabled analysis will serve healthcare providers and public health with information needed for better, more efficient decision-making, at both the clinical and population levels.

These systems and others need modernization in terms of IT and data science. Challenges include the complex organization of healthcare and public health, myriad laws and policies that vary by jurisdictions, and building in the capacity to surge and scale in times of emergency. Overcoming these challenges will require an investment and recruitment of talent. For example, one recent success is the CDC’s National Syndromic Surveillance Program, which capitalized on prior investment in BioSense, modernized its systems, partnership, and tools to create a national community of practice that made contributions to public health, not only every day but in times of local and national emergency [60,61].

Genomic testing and other precision health technologies such as digital biomarkers will increasingly present an additional source of critical data to improve health outcomes that, as yet, have not been incorporated into electronic medical record systems. How to make good use of emerging genomics data starts with the modernization of public health and healthcare data systems.

### Public health multidisciplinary strategic science

Ultimately, approaches to the application and practice of PPH will shape its impact. Two opportunities for increasing the impact of PPH practice are the use of a multidisciplinary approach and the application of a strategic science lens.

Nearly all sectors of society are increasing the use of big data and expanding possibilities for multidisciplinary approaches to PPH. Early on, PPH practice demonstrated the power of overlaying geospatial and health data as a means of capturing the “place” [2]. Data from myriad sources can be used to broaden a multidisciplinary lens both for individual and for population-level data [26]. Multi-layered use of place-based environmental data, population-level economic data, and neighborhood-level data can illuminate a range of new determinants of health outcomes. In this way, the practice of PPH will likely expand intervention recommendations from the level of individual behavior to the realm of policy and systems change [62]. This reshaping of heath determinants could advance health equity and foster multisectoral approaches to public health.

While clearly beneficial, a multidisciplinary approach to PPH will exponentially increase the volume of potentially relevant big data for public health analysis. Prioritization can be guided by a “strategic science” lens. Strategic science, loosely defined, is high-quality science that guides practice and informs policy to optimize public health impact. It starts with asking the right questions to identify what public health problems and interventions will impact public health’s explicit goals—decreasing morbidity and mortality and increasing health equity. Consideration of optimization of impact incorporates economic data, allowing for a more pragmatic approach to maximizing impact within the constraints of available resources.

### Need for ongoing innovation and workforce development

Across the public health ecosystem, organizational norms regarding data, innovation, and the workforce are shifting. As we look to harness big data to deliver PPH, commensurate investments in workforce development and innovation are needed to explore and understand its value, diverse challenges, and opportunities in today’s evolving digital healthcare environment.

In today’s digital environment, organizations that succeed in transforming and modernizing couple their human capital with systems and technology that are interoperable and accessible and provide data that support timely action [63]. As organizations rapidly move from siloed legacy infrastructure to dynamic, cloud-based environments, the enterprise becomes digitally oriented, and the capabilities of the workforce become more critical. The days of single-function work units are being eclipsed by demand for units that make strong use of emerging innovations and technologies such as artificial intelligence, machine learning, and the internet of things. Across the public health system, organizations must embrace these changes as opportunities and explore ways to augment internal capacity to support advanced tools and capabilities. Likewise, workforce development and human capital enhancement will be needed to take full advantage of the opportunities for growth.

Advanced tools, methods, and capabilities, such as big data analytics, are needed to achieve the promise of PPH and transform big data into insights, strategy, and action. In the private sector, high-performing organizations are more likely to use analytics to guide all decision-making, big or small, and at all levels of the organization [64]. Leveraging big data analytics for PPH is an opportunity in which the potential gain outweighs the harm or loss that could impact public health practice if no action is taken. Taking these and other intelligent risks requires a tolerance for failure and an expectation that innovation is not achieved through support of only successful endeavors. Organizations that invest in innovation realize that potential caveats and limitations are inherent to the process and take steps to manage the risks [65]. Realizing the value of advanced analytics for PPH will necessitate critical workforce transformation and reduction of the cultural and technical barriers to innovation and intelligent risk taking.

## Concluding remarks: PPH in the era of COVID-19

The field of PPH is clearly in its infancy, and many challenges lie ahead. Perhaps the biggest challenge of our time is the current pandemic of coronavirus disease 2019 (COVID-19). The rapid emergence of a novel coronavirus [66] has facilitated an accelerated use of the applications of “big data” tools and technologies discussed in this paper to the investigation of COVID-19. Just to illustrate, we cite the use of whole-genome sequencing [67–69] to track the virus origin and spread; detailed geographic information to track spread at the global, country, and local levels [70]; the use of smartphone-based tracking and control [71]; and rapid characterization of risk factors related to severe disease such as age, underlying medical conditions, and smoking [(72]. The role of host genomic factors is beginning to be explored [73]. Use of machine learning and data science is contributing to prediction of diagnosis and complications [74–76]. A recent commentary summarized the potential applications of emerging digital technologies [77] in augmenting public health strategies for tackling COVID-19, including public health surveillance, detection and control, and mitigation of its impact on healthcare delivery. In addition, while digital approaches to large-scale data collection can aid the investigation and control of COVID-19, ethical and social issues need to be considered, such as privacy and public trust. This will provide the opportunity to develop best practices for responsible data collection and processing globally [78]. The COVID-19 pandemic provides both a challenge and a call to action for further evolution of PPH, as new tools and technologies will begin to complement medical and public health approaches to diagnosis, treatment, control, and prevention [79]. In these challenging times, further developments in the field will require global, national, and local leadership and commitment to enhance coordination of systems; sharing, harmonization, integration, and evaluation of data; robust stakeholder engagement; and support for the infrastructure and expertise needed to achieve the promise of PPH.

## Abbreviations

CDC: Centers for Disease Control and Prevention
COVID-19: coronavirus disease 2019
FH: familial hypercholesterolemia
HBOC: hereditary breast and ovarian cancer
ILI: influenza-like illness
IT: information technology
LDL: low-density lipoprotein
PFGE: pulsed-field gel electrophoresis
PPH: precision public health
RHR: resting heart rate
